# New species and records of *Coryneum* from China

**DOI:** 10.1080/00275514.2018.1516969

**Published:** 2018-11-27

**Authors:** Ning Jiang, Hermann Voglmayr, Chengming Tian

**Affiliations:** a The Key Laboratory for Silviculture and Conservation of Ministry of Education, Beijing Forestry University, Beijing100083, China; b Division of Systematic and Evolutionary Botany, Department of Botany and Biodiversity Research, University of Vienna, Rennweg14, A-1030Vienna, Austria

**Keywords:** *Castanea*, Diaporthales, new species, *Quercus*, 3 new taxa

## Abstract

Following the abandonment of dual nomenclature and the implementation of single-name nomenclature for pleomorphic fungi, *Coryneum* was considered to have priority over *Pseudovalsa* and was recommended for use. Currently, *Coryneum* is the only genus in the family Coryneaceae (Diaporthales). However, DNA sequence data are lacking for most *Coryneum* species, and no detailed phylogenetic analyses of the genus are yet available. In the present study, fresh *Coryneum* samples were collected from chestnut (*Castanea*) and oak (*Quercus*) trees in China and morphologically compared with accepted *Coryneum* species. Based on morphological characteristics, they were identified as one known species, *Coryneum castaneicola*, and three novel species described here as *C. gigasporum, C. sinense*, and *C. suttonii*. Conidial dimensions and host association were considered major characters for species distinction. The previously unknown sexual morph of *C. castaneicola* is reported and described. A phylogenetic analysis of nuc rDNA internal transcribed spacer (ITS1-5.8S-ITS2 = ITS) and large subunit (28S) sequence data of a representative matrix of Diaporthales confirmed Coryneaceae to represent a monophyletic clade. A phylogenetic analysis of a combined sequence matrix containing the ITS-28S rDNA, the translation elongation factor 1-α (*TEF1α*), and the second largest subunit of the RNA polymerase II (*RPB2*) of the four Chinese and four additional European *Coryneum* species was performed, confirming the distinctness of these novel species.

## INTRODUCTION

Diaporthales is a diverse fungal order inhabiting plant and animal tissues, with most members being pathogens, saprobes, or endophytes (Barr 1978; Rossman et al. 2007; Senanayake et al. 2017). Coryneaceae, formerly commonly known as Pseudovalsaceae, are characterized by having black perithecia, often immersed in wood, asci that deliquesce at maturity, and an asexual morph with transversely distoseptate brown conidia (Sutton 1975; Rossman et al. 2007; Senanayake et al. 2017). Recent molecular phylogenetic analyses of Diaporthales showed that Coryneaceae represents a monophyletic family among Diaporthales (Voglmayr and Jaklitsch 2014; Senanayake et al. 2017; Voglmayr et al. 2017; Fan et al. 2018), including only the single genus *Coryneum*. However, few studies of *Coryneum* at the species level have been undertaken.

*Coryneum* was first described based on *C. umbonatum* (Nees von Esenbeck 1816), which is the asexual morph of *Pseudovalsa longipes* (Sutton 1975). The type species of *Pseudovalsa, P. lanciformis*, is the sexual morph of another *Coryneum* species, *C. brachyurum* (Sutton 1975), now *C. lanciforme*. Therefore, in single-name nomenclature, the two genera become synonyms. Based on priority and the need for fewer new combinations, Rossman et al. (2015) recommended the genus name *Coryneum* for use rather than *Pseudovalsa*. Also at the family level, the older name Coryneaceae has priority over Pseudovalsaceae (Senanayake et al. 2017).

In his extensive monograph, Sutton (1975) transferred numerous species of *Coryneum* to other genera and accepted 19 species and one variety. Subsequently, *Coryneum arausiacum* (Senanayake et al. 2017), *C. gregoryi* (Sutton 1986), *C. pruni* (Wijayawardene et al. 2016), *C. quercinum* (Muthumary and Sutton 1986), and *C. terrophilum* (Sutton 1986) were added as new species or combinations. *Coryneum* species are generally considered highly host specific, especially occurring on hardwood trees such as those in the Betulaceae and Fagaceae (Sutton 1975). A summary of conidial sizes and host information for currently accepted *Coryneum* species is listed in TABLE 1. Few sequence data are available for most described *Coryneum* species, and considering that host identifications may be incorrect and that many geographical areas are still insufficiently studied, synonymies and actual numbers of *Coryneum* species are still unsettled.

**Table 1.** Hosts, conidial sizes, and numbers of distosepta of currently accepted *Coryneum* species.

Species	Host	Conidial size (μm)	No. of distosepta	Reference
*Coryneum arausiacum*	*Quercus*	42–56 × 13–16	4–5	Senanayake et al. (2017)
*C. betulinum*	*Betula*	31–36 × 14–17	4–5	Sutton (1975)
*C. calophylli*	*Calophyllum inophyllum*	38–48 × 12.5–14.5	5–6	Sutton (1975)
*C. carpinicola*	*Carpinus*	50–68 × 8–11	7–11	Sutton (1975)
*C. castaneicola*	*Castanea*	56–80 × 9.5–13	5–8	Sutton (1975), this study
*C. cesatii*	*Aesculus*	80–90 × 13–15	6–7	Sutton (1975)
*C. clusiae*	*Clusia*	30–40 × 20–30	3–5	Sutton (1975)
*C. compactum*	*Ulmus*	40–58 × 15–21	4–6	Sutton (1975)
*C. depressum*	*Quercus*	44–53 × 19–23	4–6	Sutton (1975)
*C. elevatum*	*Quercus*	56–69 × 24–28	5–7	Sutton (1975)
*C. gigasporum*	*Castanea mollissima*	88–117 × 18–23	7–9	This study
*C. gregoryi*	*Eucalyptus globulus*	32.5–43 × 12–16	5–9	Sutton and Sharma (1983)
*C. japonicum*	*Quercus*	45–60 × 11–12	5–7	This study
*C. lanciforme* (= *C. brachyurum*)	*Betula*	45–53 × 16–18	4–6	Sutton (1975)
*C. megaspermum*	*Quercus*	73–97 × 13–16	7–11	Sutton (1980)
*C. megaspermum* var. *cylindricum*	*Quercus*	100–125 × 10–13	7–8	Sutton (1975)
*C. modonium*	*Castanea*	50–71 × 14–19	5–8	Sutton (1975)
*C. neesii*	*Quercus*	68–82 × 18–22	6–8	Sutton (1975)
*C. pruni*	*Prunus*	14–23 × 5.5–9	4–5	Wijayawardene et al. (2016)
*C. psidii*	*Psidium guajava*	25–40 × 14–17	5–6	Sutton (1975)
*C. pyricola*	*Pyrus*	61–70 × 24–32	5–7	Sutton (1975)
*C. quercinum*	*Quercus*	45–60 × 14–16	6–7	Muthumary and Sutton (1986)
*C. sinense*	*Quercus serrata*	50–76 × 13–17	5–7	This study
*C. stromatoideum*	*Tsuga canadensis*	105–180 × 16–20	9–17	Sutton (1975)
*C. suttonii*	*Castanea mollissima*	60–76 × 10–14.5	4–5	This study
*C. sydowianum*	*Alnus incaca*	50–58 × 14–17	5–6	Sutton (1975)
*C. terrophilum*	Soil	25–55 × 15–24	3–7	Sutton and Sharma (1983)
*C. umbonatum*	*Quercus*	57–72 × 13–16	5–7	Sutton (1975)

Species of *Castanea* and *Quercus* (Fagaceae), which comprise economically as well as ecologically important trees in temperate to subtropical forest ecosystems, are hosts for diaporthalean fungi of various lineages. *Cryphonectria parasitica* is the most important canker pathogen of chestnut worldwide, but it can also incidentally infect oaks (Rigling and Prospero 2018). *Diaporthe eres* was reported from leaves of *Castanea mollissima* (Gong et al. 2017). *Gnomoniopsis smithogilvyi* (= *G. castaneae*) is one of the causal agents of chestnut fruit rot (Shuttleworth et al. 2016). *Amphiporthe leiphaemia* and *Caudospora taleola* are common but usually weak canker pathogens of oaks in Europe (Phillips and Burdekin 1992). In addition, several *Coryneum* species occur on chestnut and oak trees (Sutton 1975).

Recently, relationships within and amongst genera and families of Diaporthales were studied based on multigene sequence data (Sogonov et al. 2008; Mejía et al. 2011a, 2011b; Crous et al. 2012; Voglmayr et al. 2012, 2017; Walker et al. 2012a, 2014a, 2014b; Gomes et al. 2013; Udayanga et al. 2014, 2015; Alvarez et al. 2016; Fan et al. 2016, 2018; Senanayake et al. 2017), but for most diaporthalean lineages only nuc rDNA internal transcribed spacer (ITS1-5.8S-ITS2 = ITS) and large subunit (28S) sequence data are available. Presently, identification of *Coryneum* species using sequences is difficult because of the absence of ex-type strains and ITS sequence data. Within Coryneaceae, ITS, 28S, translation elongation factor 1-α (*TEF1α*) sequences are available only for two isolates identified as *C. arausiacum*; for the second largest subunit of the RNA polymerase II (*RPB2*) and 28S, sequences are available for single accessions of three additional species, namely, *C. depressum, C. modonium*, and *C. umbonatum*. The aim of the present study was to initiate taxonomic work on *Coryneum* combining morphology and multigene phylogeny. Fresh specimens of *Coryneum* from *Castanea* and *Quercus* hosts were collected in western China, and pure cultures were isolated from single conidia or ascospores. Culturing proved challenging because of slow colony growth on potato dextrose agar (PDA) and malt extract agar (MEA). In addition, multigene data were generated for four well-characterized, common European *Coryneum* species, including the generic types of *Coryneum* and its synonym *Pseudovalsa*. As a result of our analyses, four *Coryneum* species are described and illustrated from China, three of which represent new species, and a new connection of sexual and asexual morphs of one species is described based on sequence data.

## MATERIALS AND METHODS

### Isolation

Fresh specimens of *Coryneum* were collected from branches of *Castanea mollisima* and *Quercus serrata* during our survey in Shaanxi Province, China. Single conidial and ascospore isolates were established by removing a mucoid spore mass from conidiomata or ascomata and spreading the suspension on the surface of 1.8% potato dextrose agar (PDA; 200 g potatoes, 20 g dextrose, 20 g agar per L). After inoculation, agar plates were incubated at 25 C to induce germination of spores. Single germinating spores were then transferred to new plates under a dissecting microscope with a sterile needle (Fan et al. 2018). Specimens and isolates were deposited in the Museum of Beijing Forestry University (BJFC). Axenic cultures are maintained in the China Forestry Culture Collection Center (CFCC).

### Morphological observations

Species identification was based on morphological characters of the conidiomata and ascomata produced on infected plant tissues. Cross-sections were prepared by hand using a double-edge blade under a Leica stereomicroscope (M205 FA; Wetzlar, Germany). Photomicrographs were captured with a Nikon Eclipse 80i microscope equipped with a Nikon digital sight DS-Ri2 high definition color camera, using differential interference contrast (DIC) illumination and the Nikon software NIS-Elements D Package 3.00 (Tokyo, Japan). Measurements of ascospores and conidia are reported as maximum and minimum in parentheses and the range representing the mean ± standard deviation of the number of measurements given in parentheses. Cultural characteristics of isolates incubated on PDA in the dark at 25 C were recorded.

### DNA extraction, PCR, and sequencing

Genomic DNA was extracted from axenic living cultures with cellophane using a modified cetyltrimethylammonium bromide (CTAB) method (Doyle and Doyle 1990) or from freeze-dried liquid cultures (Voglmayr and Jaklitsch 2011) using the DNeasy Plant Mini Kit (Qiagen, Hilden, Germany). To amplify the ITS, we used primers ITS1 and ITS4 (White et al. 1990); the 28S, the primers LR0R and LR5 (Moncalvo et al. 1995; Vilgalys and Hester 1990); *TEF1α*, the primers EF1-688F or EF1-728F and EF1-986R or TEF1-LLErev (Carbone and Kohn 1999; Jaklitsch et al. 2006; Alves et al. 2008); and *RPB2*, the primers fRPB2-5F and fRPB2-7cR (Liu et al. 1999) or dRPB2-5f and dRPB2-7r (Voglmayr et al. 2016). In some instances, the ITS-28S region was amplified and sequenced as a single fragment with primers V9G (de Hoog and Gerrits van den Ende 1998) and LR5, with ITS4 and LR3 (Vilgalys and Hester 1990) as additional sequencing primers. The polymerase chain reaction (PCR) assay was conducted as described by Fan et al. (2018). Amplification products were visually checked by electrophoresis in 2% agarose gels. DNA sequencing was performed using an ABI PRISM 3730xl DNA analyzer (Carlsbad, California) with BigDye Terminator 3.1 kit (Invitrogen, Foster City, California) at the Shanghai Invitrogen Biological Technology Company Limited (Beijing, China) or at the Department of Botany and Biodiversity Research of the University of Vienna.

### Phylogenetic analyses

For the phylogenetic placement of the *Coryneum* taxa included in our analyses, a representative ITS-28S matrix including 58 members of all currently accepted families of Diaporthales was produced, with two species of Magnaporthales (*Nakataea oryzae, Pyricularia oryzae*) selected as outgroups. For detailed investigations of species relationships and delimitation within *Coryneum* species, a combined matrix of three loci (ITS-28S rDNA, *RPB2, TEF1α*) was produced for phylogenetic analyses, with two species of Stilbosporaceae (*Stilbospora macrospora* and *Stegonsporium pyriforme*; Voglmayr and Jaklitsch 2014) selected as outgroups. The GenBank accession numbers of sequences used in these analyses are given in TABLE 2.

**Table 2.** Strains and NCBI GenBank accession numbers used in this study.

Species	Strain/Specimen	Host	GenBank accession numbers			
			ITS	28S	*TEF1α*	*RPB2*
*Apiosporopsis carpinea*	CBS 771.79	*Carpinus betulus*	NA	AF277130		
*Apiosporopsis* sp.	Masuya 11Af2-1	*Alnus firma*	NA	AB669034		
*Apoharknessia insueta*	CBS 111377	*Eucalyptus pellita*	JQ706083	AY720814		
*Asterosporium asterospermum*	MFLU 15-3555	*Fagus sylvatica*	NA	MF190062		
*Auratiopycnidiella tristaniopsidis*	CBS 132180 = CPC 16371	*Tristaniopsis laurina*	JQ685516	JQ685522		
*Chiangraiomyces bauhiniae*	MFLUCC 17-1669	*Bauhinia* sp.	MF190118	MF190064		
*Coniella straminea*	CBS 149.22 = CPC 3932	*Fragaria* sp.	AY339348	AF362569		
*Coniella wangiensis*	CBS 132530 = CPC 19397	*Eucalyptus* sp.	JX069873	JX069857		
*Coryneum arausiacum*	MFLUCC 13-0658	*Quercus* sp.	MF190120	MF190066		
*Coryneum arausiacum*	MFLUCC 15-1110	*Quercus* sp.	MF190121	MF190067		
***Coryneum castaneicola***	CFCC 52315	*Castanea mollissima*	MH683551	MH683559	MH685731	MH685723
***Coryneum castaneicola***	CFCC 52316	*Castanea mollissima*	MH683552	MH683560	MH685732	MH685724
***Coryneum depressum***	D202	*Quercus petraea*	MH674330	MH674330	MH674338	MH674334
*Coryneum depressum*	AR 3897	*Quercus cerris*	NA	EU683074		
***Coryneum gigasporum***	CFCC 52319	*Castanea mollissima*	MH683557	MH683565	MH685737	MH685729
***Coryneum gigasporum***	CFCC 52320	*Castanea mollissima*	MH683558	MH683566	MH685738	MH685730
***Coryneum lanciforme***	D215	*Betula pubescens*	MH674332	MH674332	MH674340	MH674336
***Coryneum modonium***	D203	*Castanea sativa*	MH674331	MH674331	MH674339	MH674335
*Coryneum modonium*	AR 3558	*Castanea sativa*	NA	EU683073		
***Coryneum sinense***	CFCC 52452	*Quercus serrata*	MH683553	MH683561	MH685733	MH685725
***Coryneum sinense***	CFCC 52453	*Quercus serrata*	MH683554	MH683562	MH685734	MH685726
***Coryneum suttonii***	CFCC 52317	*Castanea mollissima*	MH683555	MH683563	MH685735	MH685727
***Coryneum suttonii***	CFCC 52318	*Castanea mollissima*	MH683556	MH683564	MH685736	MH685728
***Coryneum umbonatum***	D201	*Quercus robur*	MH674329	MH674329	MH674337	MH674333
*Coryneum umbonatum*	AR 3541	*Quercus cerris*	NA	EU683072		
*Coryneum umbonatum*	MFLUCC 15-1110	*Quercus* sp.	MF190121	MF190067		
*Coryneum umbonatum*	MFLUCC 13-0658	*Quercus* sp.	MF190120	MF190066		
*Cryphonectria macrospora*	AR 3444 = CBS 109764	*Quercus mongolica*	EU199182	AF408340		
*Cryphonectria parasitica*	ATCC 38755	*Castanea dentata*	AY141856	EU199123		
*Cryptosporella hypodermia*	AR 3552 = CBS 122593	*Ulmus minor*	EU199181	AF408346		
*Cytospora chrysosperma*	CFCC 89600	*Sophora japonica*	KR045623	KR045623		
*Dendrostoma mali*	CFCC 52102	*Malus spectabilis*	MG682072	MG682012		
*Diaporthe eres*	AR 3538 = CBS 109767	*Acer campestre*	KC343075	AF408350		
*Diaporthosporella cercidicola*	CFCC 51994	*Cercis chinensis*	KY852492	KY852515		
*Diaporthostoma machili*	CFCC 52100	*Machilus leptophylla*	MG682080	MG682020		
*Disculoides eucalypti*	CPC 17650	*Eucalyptus* sp.	JQ685517	JQ685523		
*Ditopella ditopa*	AR 3423 = CBS 109748	*Alnus glutinosa*	EU199187	EU199126		
*Erythrogloeum hymenaeae*	CPC 18819	*Hymenaea courbaril*	JQ685519	JQ685525		
*Gnomonia gnomon*	CBS 199.53	*Corylus avellana*	AY818956	AF408361		
*Harknessia eucalypti*	CBS 342.97	*Eucalyptus regnans*	AY720745	AF408363		
*Harknessia molokaiensis*	AR 3578 = CBS 109779	*Eucalyptus robusta*	NA	AF408390		
*Hercospora tiliae*	AR 3526 = CBS 109746	*Tilia tomentosa*	NA	AF408365		
*Juglanconis appendiculata*	D96	*Juglans nigra*	KY427139	KY427139		
*Juglanconis juglandina*	ME23	*Juglans nigra*	KY427150	KY427150		
*Lamproconium desmazieri*	MFLUCC 15-0870	*Tilia tomentosa*	KX430134	KX430135		
*Lasmenia* sp.	CBS 124123	*Nephelium lappaceum*	GU797406	JF838338		
*Macrohilum eucalypti*	CPC 10945	*Eucalyptus* sp.	DQ195781	DQ195793		
*Melanconiella ellisii*	BPI 878343	*Carpinus caroliniana*	JQ926271	JQ926271		
*Melanconiella spodiaea*	MSH	*Carpinus betulus*	JQ926298	JQ926298		
*Melanconis betulae*	CFCC 50471	*Betula albosinensis*	KT732952	KT732971		
*Melanconis stilbostoma*	CFCC 50475	*Betula platyphylla*	KT732956	KT732975		
*Nakataea oryzae*	CBS 243.76	NA	KM484861	DQ341498		
*Pachytrype princeps*	Rogers S	NA	NA	FJ532382		
*Paradiaporthe artemisiae*	MFLUCC 14-0850	*Artemisia* sp.	MF190155	MF190100		
*Prosopidicola mexicana*	CBS 113530	*Prosopis glandulosa*	AY720710	NA		
*Pseudomelanconis caryae*	CFCC 52110	*Carya cathayensis*	MG682082	MG682022		
*Pseudoplagiostoma eucalypti*	CBS 124807	*Eucalyptus urophylla*	GU973512	GU973606		
*Pseudoplagiostoma oldii*	CBS 115722	*Eucalyptus camaldulensis*	GU973535	GU973610		
*Pyricularia grisea*	Ina168	NA	AB026819	AB026819		
*Rossmania ukurunduensis*	AR 3484	*Acer ukurunduense*	NA	EU683075		
*Stegonsporium pyriforme*	CBS 124487	*Acer heldreichii*	KF570160	KF570160		
*Stilbospora macrosperma*	CBS 121883	*Carpinus betulus*	JX517290	JX517299		
*Sydowiella fenestrans*	AR 3777 = CBS 125530	*Chamerion angustifolium*	JF681956	EU683078		
*Synnemasporella aculeans*	CFCC 52094	*Rhus chinensis*	MG682086	MG682026		
*Synnemasporella toxicodendri*	CFCC 52097	*Toxicodendron sylvestre*	MG682089	MG682029		

*Note*. Strains from this study are in bold. NA refers to the phylogenetic analysis of the ITS-LSU matrix, in cases where only one of the two sequence regions (either ITS or LSU) was available. In the tef1 and rpb2 column, only the sequences used for the multigene (ITS, LSU, tef1, rpb2) analyses are listed – empty spaces mean that the corresponding taxa were not considered for the multigene analyses, irrespective whether a sequence is available or not.

Sequences from this study and reference sequences obtained from GenBank (TABLE 2) were aligned and edited manually using MEGA6 (Tamura et al. 2013). The alignments were concatenated for phylogenetic analyses. Maximum parsimony (MP) analyses were conducted with PAUP 4.0b10 (Swofford 2003), using 1000 heuristic search replicates with random additions of sequences with the tree bisection and reconnection (TBR) branch swapping algorithm (MULTREES option in effect, steepest descent option not in effect). All molecular characters were unordered and given equal weight; analyses were performed with gaps treated as missing data; the COLLAPSE command was set to minbrlen, and in the ITS-28S analyses, maxtrees was set to 5000. All equally parsimonious trees found were saved in the MP analyses. Other calculated parsimony scores were tree length (TL), consistency index (CI), retention index (RI), and rescaled consistency (RC). MP bootstrap analyses with 1000 replicates were performed in the same way, with 10 rounds of replicates of heuristic search with random addition of sequences and subsequent TBR branch swapping during each bootstrap replicate. To check for congruence amongst the three loci for evaluation whether they meet the genealogical concordance phylogenetic species recognition (GCPSR) concept (Taylor et al. 2000), MP bootstrap analyses were also performed separately for the ITS-28S, *RPB2*, and *TEF1α* matrices.

Maximum likelihood (ML) analyses of the ITS-28S matrix were performed with PhyML 7.2.8, with a GTR site substitution model, including a gamma-distributed rate heterogeneity and a proportion of invariant sites (Guindon et al. 2010). Branch support was evaluated with a bootstrapping (BS) method with 1000 replicates. ML analyses of the three-locus matrix were done with RAxML (Stamatakis 2006) as implemented in raxmlGUI 1.3 (Silvestro and Michalak 2012), using the ML + rapid bootstrap setting and the GTRGAMMA substitution model with 1000 bootstrap replicates. In the ML analyses, the combined three-locus matrix was partitioned for the individual gene regions, and substitution model parameters were calculated separately for each. Bayesian inference (BI) of the ITS-28S matrix was performed using MrBayes 3.1.2 (Ronquist and Huelsenbeck 2003), implementing the GTR+I+G model according to the results of MrModeltest. Two Markov chain Monte Carlo (MCMC) chains were run from random trees for 1 million generations and stopped when average standard deviation of split frequencies fell below 0.01. Trees were saved each 1000 generations. The first 25% of trees were discarded as the burn-in phase of each analysis, and the posterior probabilities (BPPs) were calculated from the remaining trees. The alignments and trees are deposited in TreeBASE (study no. S22414). Taxonomic novelties were deposited in MycoBank (Crous et al. 2004).

## RESULTS

**Figure 1.**
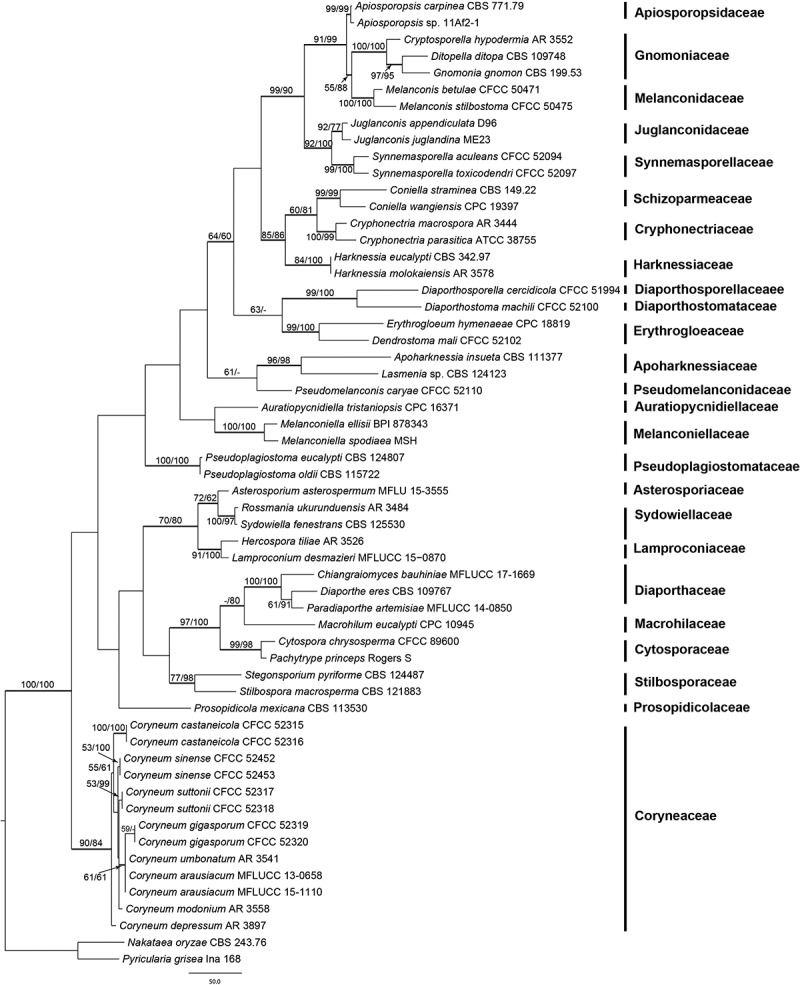
Phylogram showing one of 570 most parsimonious trees of 2620 steps revealed by an analysis of the combined ITS-28S matrix of selected Diaporthales. Values above or below the branches indicate maximum parsimony and maximum likelihood bootstrap support. Thickened branches represent posterior probabilities above 0.90 from Bayesian inference. Bar: 50 nucleotide substitutions.

The final combined ITS-28S matrix comprised 1564 alignment characters. Of these, 937 characters were constant, 126 variable characters were parsimony uninformative, and 501 characters were parsimony informative. The MP analyses of the ITS-28S matrix resulted in 570 equally most parsimonious trees, with the first tree (TL = 2620, CI = 0.418, RI = 0.643, RC = 0.269) shown in FIG. 1. The phylogenetic trees obtained from ML and BI analyses with the MCMC algorithm were consistent with the MP tree shown in FIG. 1. Isolates of *Coryneum* species from this study and previous studies grouped together in a distinct Coryneaceae clade within Diaporthales, which is separate from all other families and receives high support (ML/MP/BI = 92/93/1). This is also supported by morphological characters. However, phylogenetic relationships within *Coryneum* remain unresolved because of low or insignificant support, indicating insufficient phylogenetic information of the ITS-28S sequence data.

**Figure 2.**
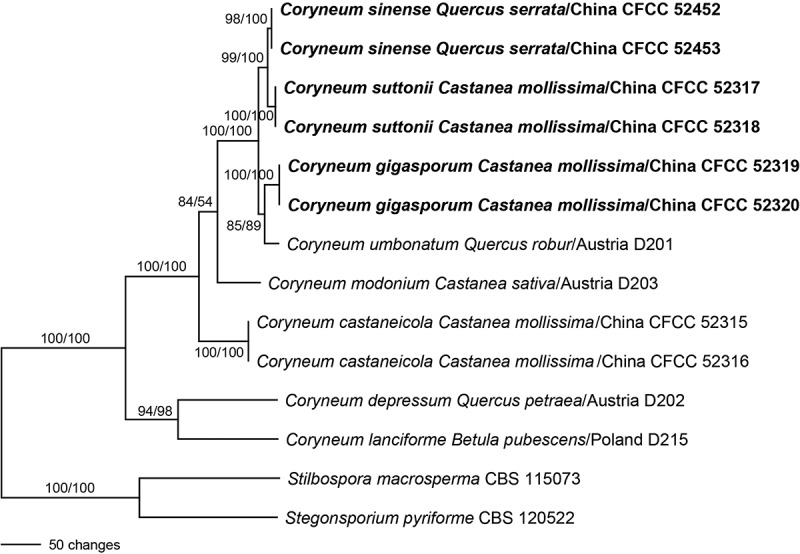
Phylogram showing the single most parsimonious tree of 1386 steps revealed by an analysis of the combined ITS-28S-*RPB2-TEF1α* matrix of *Coryneum*. MP and ML bootstrap support values above 50% are given at the first and second positions, respectively, above or below the branches. Hosts, countries, and strain/culture numbers are given following the names of the taxa; new species described in the present study are formatted in bold. Bar: 50 nucleotide substitutions.

The three-locus multigene matrix contained 3953 characters (1500 from ITS-28S, 1079 from *RPB2*, and 1374 from *TEF1α*). Of these, 3002 characters were constant, 387 variable characters were parsimony uninformative, and 564 parsimony informative (101 from ITS-28S, 231 from *RPB2*, and 232 from *TEF1α*). The MP analyses resulted in a single MP tree of 1386 steps (CI = 0.852, RI = 0.796, RC = 0.678), which is shown in FIG. 2. Tree topology of the best tree revealed by the ML analyses was identical to that of the MP tree (not shown). Conversely to the ITS-28S analyses, in the three-locus multigene analyses *Coryneum* species as well as most internal nodes receive high to maximum support, demonstrating a substantial increase of phylogenetic resolution by the addition of *RPB2* and *TEF1α* sequences.

The separate MP bootstrap analyses of the ITS-28S, *RPB2*, and *TEF1α* matrices revealed compatible topologies of the three loci, except for a moderately (85%, *RPB2*) to highly (100%, *TEF1α*) supported *C. suttonii*–*C. sinense* clade in conflict with a sister-group relationship of the *C. sinense* clade to the *C. suttonii–C. umbonatum–C. gigasporum* clade in ITS-28S, which, however, is only very poorly supported (51%) (SUPPLEMENTARY FIG. 1). The phylogenies of the three loci are therefore considered congruent, meeting the GCPSR concept.

## TAXONOMY

**Figure 3.**
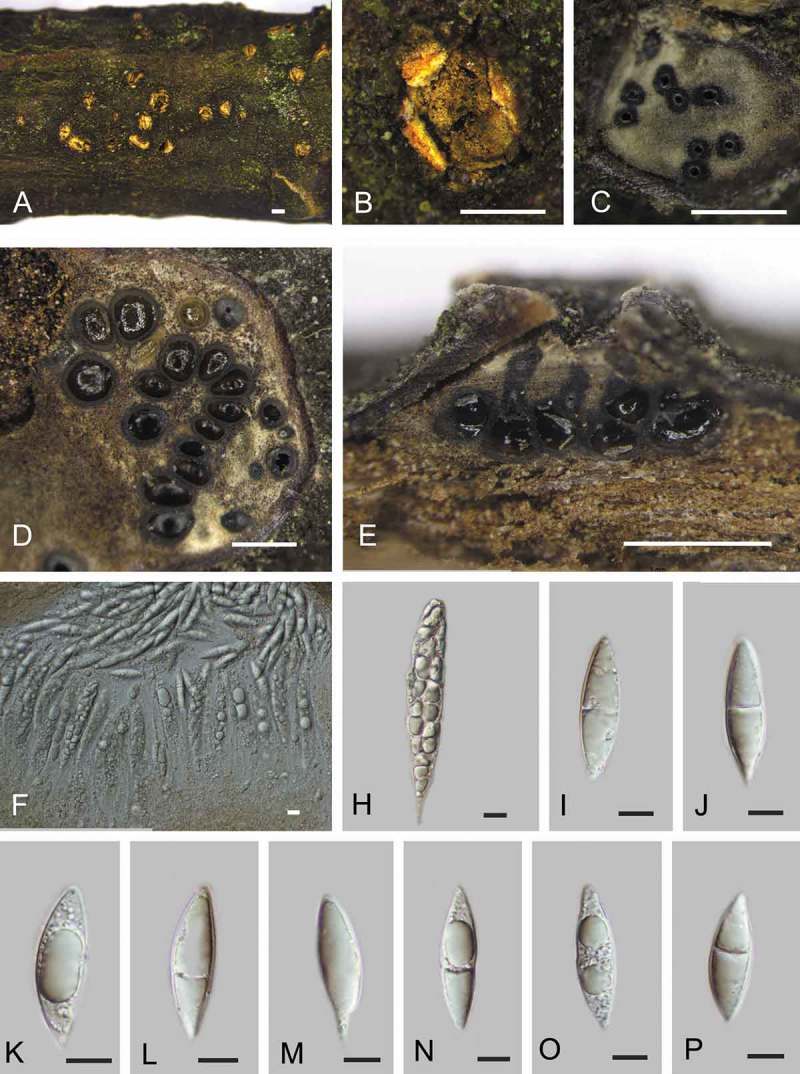
Sexual morph of *Coryneum castaneicola* from *Castanea mollissima* (BJFC-S1419). A, B. Ectostromatic discs in face view. C. Transverse section below ectostromatic disc. D. Pseudostroma in transverse section, showing perithecia and gray entostroma. E. Longitudinal sections through pseudostromata. F. Asci. I–P. Ascospores. Bars: A–E = 0.5 mm; F–P = 10 μm.

**Figure 4.**
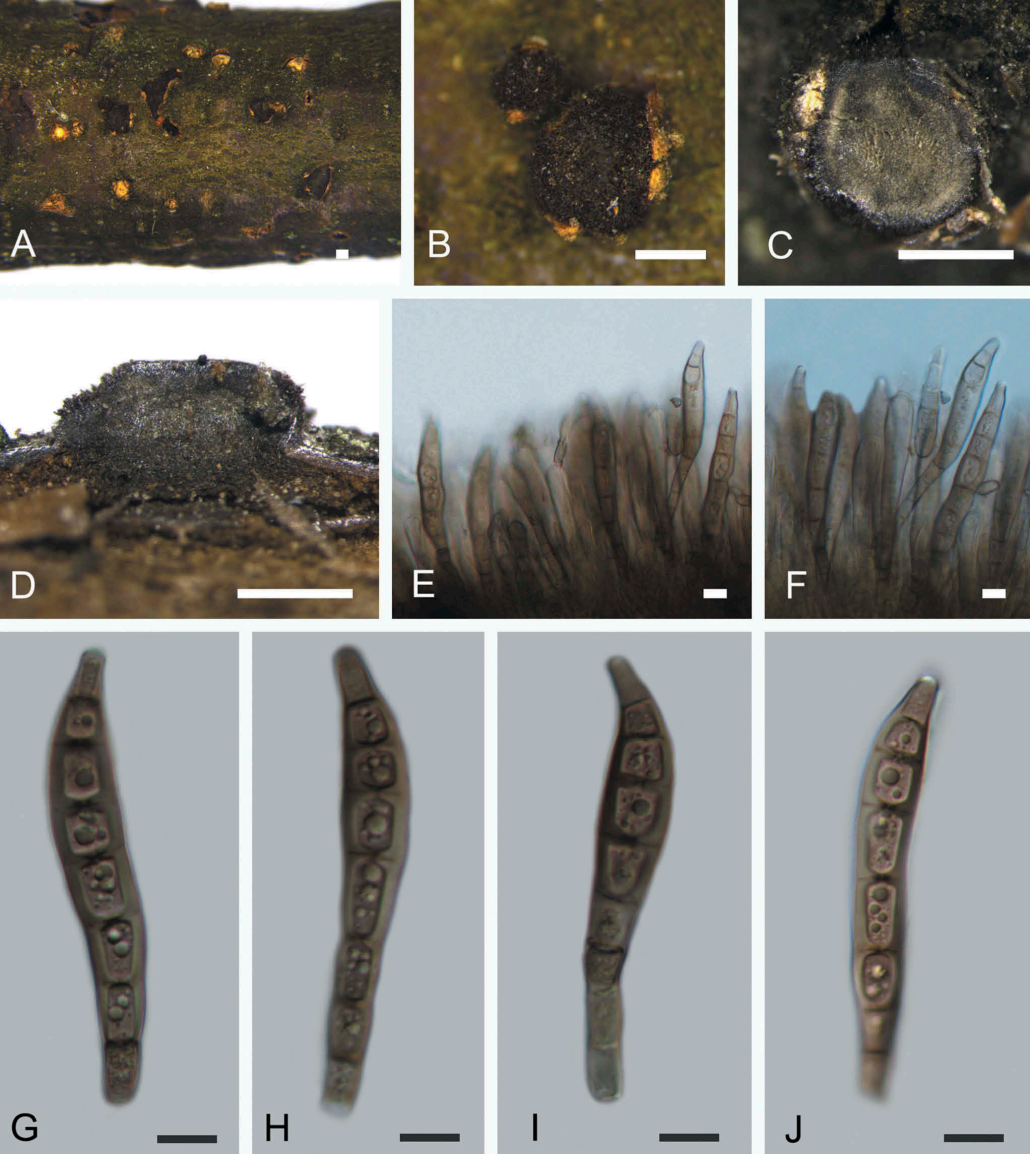
Asexual morph of *Coryneum castaneicola* from *Castanea mollissima* (BJFC-S1419). A, B. Conidiomata on natural substrate in surface view. C. Transverse section through conidioma. D. Longitudinal section through conidioma. E, F. Conidiophores. G–J. Conidia. Bars: A–D = 0.5 mm; E–J = 10 μm.

*Coryneum castaneicola* Berk. & M.A. Curtis, Grevillea 2:154. 1874. FIGS. 3, 4

*Sexual morph*: Pseudostromata 0.3–1.5 mm diam, typically distinct, circular, without perithecial bumps, containing up to 25 perithecia embedded in a well-developed entostroma. Ectostromatic disc distinct, circular, orange, 0.3–0.6 mm diam. Central column and entostroma gray. Ostioles inconspicuous and often invisible at surface of ectostromatic disc. Perithecia (300–)350–700(–750) μm diam (n = 20), globular, somewhat flattened at base with black neck. Asci 180–250 × 25–45 μm ($\overset{ˉ}{x}$ = 225 × 35 μm, n = 10), 8-spored, unitunicate, clavate, shortly pedicellate, apically rounded, with an inconspicuous apical ring. Ascospores 36–43(–44.5) × (9.5–)10.5–12(–13) μm, L/W = (2.8–)3.1–3.5(–3.8) (n = 50), 2–3-seriate, fusiform, ends pointed, uniseptate or aseptate, not constricted at septa, hyaline, guttulate, smooth-walled.

*Asexual morph*: Conidiomata acervular, 0.2–2.5 mm wide, 0.2–2.0 mm high ($\overset{ˉ}{x}$ = 1.8 × 1.1 mm, n = 20), solitary, erumpent through outer periderm layers of host, scattered, surface tissues above slightly domed. Conidiophores 50–80 μm long, 4–7 μm wide ($\overset{ˉ}{x}$ = 64 × 6 μm, n = 20), branched at base, cylindrical, septate, hyaline at apex, pale brown at base. Conidiogenous cells holoblastic, integrated, indeterminate, cylindrical, expanding toward apices, pale brown, smooth, with 0–1 percurrent extensions. Conidia (56–)65–73(–79) × (9.5–)10.5–12(–12.5) μm, L/W = (5.6–)5.9–6.3(–6.6) (n = 50), variable in shape, curved, broadly fusiform to fusiform, cylindrical or clavate, dark brown, smooth-walled, 6–7-distoseptate, apical cell with a hyaline tip, truncate and black at base.

*Culture characters*: On PDA at 25 C, colonies growing slowly and unevenly, reaching 70 mm diam within 30 d, gradually becoming brownish gray to dark gray in color with scant cottony aerial mycelium, asexual morphs developed after 2 mo.

*Habitat and host range*: Dead corticated branches of *Castanea* spp.

*Additional specimens examined*: CHINA. SHAANXI PROVINCE: Ankang City, Xiangxidong Garden, 32°40′32.51″N, 109°18′57.36″E, 1079 m above sea level (asl), sexual and asexual morphs on branches of *Castanea mollissima, N. Jiang*, 1 Jul 2017 (BJFC-S1419; culture CFCC 52315 grown from conidium, culture CFCC 52316 grown from ascospore); Xiangxidong Garden, 32°40′32.51″N, 109°18′’57.36″E, 1079 m asl, sexual and asexual morphs on branches of *C. mollissima, N. Jiang*, 1 Jul 2017 (BJFC-S1420).

*Notes*: Two specimens of *Coryneum* collected from chestnut branches in China were identified as *C. castaneicola* based on their morphology (Sutton 1975). Cultures and sequences were obtained from both sexual and asexual morphs from the same specimen, which confirms a holomorph connection. *Coryneum castaneicola* was described and was previously only known from North America, where it has been recorded from species of *Castanea* (Sutton 1975; Farr and Rossman 2018). The conidial dimensions (56–79 × 9.5–12.5 μm in CFCC 52315 vs. 65.2–73.4 × 10.6–11.9 μm in the type slide IMI 180179, fide Sutton 1975, 1980) and numbers of distoseptate cells (6–7-distoseptate in CFCC 52315 vs. 5–7-distoseptate in the type slide, fide Sutton 1975, 1980) match perfectly, and we therefore consider our Chinese specimens to be conspecific with the North American type. However, sequences from North American material are necessary for final confirmation of conspecificity.

The sexual morph of *C. castaneicola*, described here for the first time, has ascospores that are similar in shape, color, and septation to those of *C. modonium*, reported from species of *Castanea* in Asia, Europe, and North America (Kobayashi 1970; Sutton 1975). However, the ascospores of the latter are shorter (23–38 × 8–13.5 µm; see Wehmeyer 1941; Ellis and Ellis 1997) than those of *C. castaneicola* (36–44.5 × 9.5–13 µm). *Coryneum modonium* also differs by straight fusiform conidia, which are shorter but wider ((44–)50–71(–75) × 14–19(–22) µm) than those of *C. castaneicola*, which is also well characterized by apically distinctly curved conidia (Sutton 1975).

The identity of the Japanese collections on *Castanea crenata* described and illustrated by Kobayashi (1970) as *Pseudovalsella modonia*, a synonym of *C. modonium*, is unclear and requires detailed investigations. Whereas size and shape of the ascospores match European collections of *C. modonium*, their conidial width was reported as even narrower than in *C. castaneicola* (6.5–10 µm; Kobayashi 1970). Therefore, the Japanese collections may represent another undescribed species.

**Figure 5.**
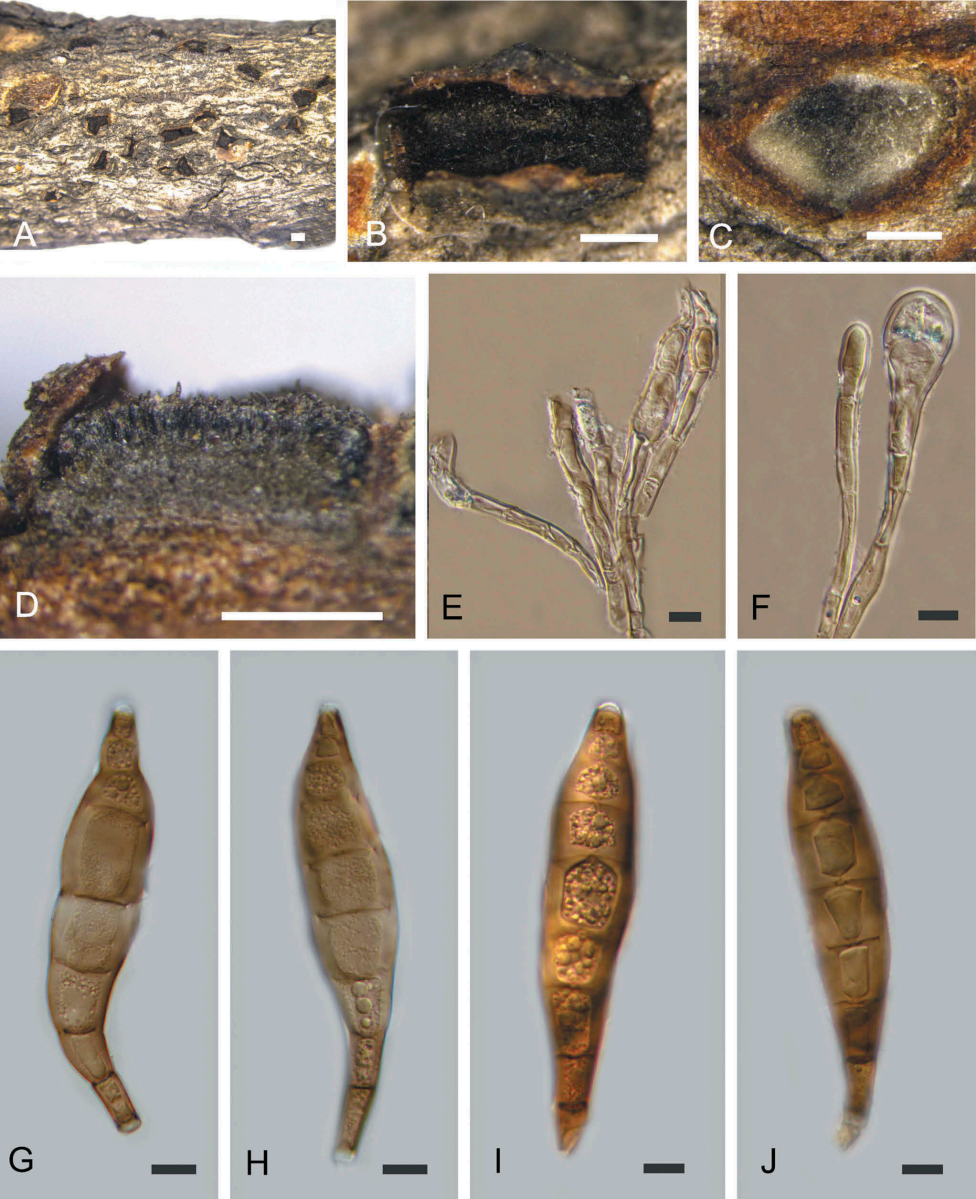
Morphology of *Coryneum gigasporum* from *Castanea mollissima* (BJFC-S1425, holotype). A, B. Conidiomata on natural substrate in surface view. C. Transverse section through conidioma. D. Longitudinal section through conidioma. E. F. Conidiophores. G–J. Conidia. Bars: A–D = 0.5 mm; E–J = 10 μm.

***Coryneum gigasporum*** C.M. Tian, Voglmayr & N. Jiang, sp. nov. FIG. 5

MycoBank MB824594.

*Typification*: CHINA. SHAANXI PROVINCE: Shangluo City, chestnut plantation, 33°38′21.03″N, 109°08′45.22″E, 2602 m asl, on branches of *Castanea mollissima, N. Jiang*, 8 Jul 2017 (**holotype** BJFC-S1425). Ex-type culture: CFCC 52319.

*Etymology: gigasporum* (Latin), named after the very large conidia.

*Sexual morph*: Not observed.

*Asexual morph*: Conidiomata acervular, 0.8–1.5 mm wide, 0.5–1.0 mm high ($\overset{ˉ}{x}$ = 1.0 × 0.7 mm, n = 20), solitary, erumpent through outer periderm layers of host, scattered, surface tissues above slightly domed. Conidiophores 50–90 μm long, 3–6 μm wide ($\overset{ˉ}{x}$ = 55 × 5 μm, n = 20), unbranched, cylindrical, septate, hyaline at apex, pale brown at base. Conidiogenous cells holoblastic, integrated, indeterminate, cylindrical, expanding toward apices, hyaline to pale brown, smooth, with 0–1 percurrent extensions. Conidia (88–)93–108(–117) × (18–)19–21(–23) μm, L/W = (4.2–)4.6–5.4(–5.6) (n = 50), slightly curved or not, clavate, dark brown, smooth-walled, 7–9-distoseptate, apical cell with a hyaline tip, truncate and black at base.

*Culture characters*: On PDA at 25 C, colonies growing slowly and symmetrically, reaching 70 mm diam within 30 d, gradually becoming brownish gray in color with scant cottony aerial mycelium, asexual morphs developed after 2 mo.

*Habitat and host range*: Dead corticated branches of *Castanea mollissima*.

*Additional specimen examined*: CHINA. SHAANXI PROVINCE: Shangluo City, chestnut plantation, 33°38′21.03″N, 109°08′45.22″E, 2602 m asl, on branches of *C. mollissima, N. Jiang*, 8 Jul 2017 (BJFC-S1426; living culture CFCC 52320).

*Notes*: Conidial size and shape are a main character for species distinction in *Coryneum* (Sutton 1975). *Coryneum gigasporum* is unique for its large conidial size (88–117 × 18–23 μm) within the genus. The two other *Coryneum* species with very long conidia, *C. megaspermum* var. *cylindricum* from *Quercus* and *C. stromatoideum* from *Tsuga canadensis*, differ from *C. gigasporum* by longer and narrower conidia (TABLE 1).

**Figure 6.**
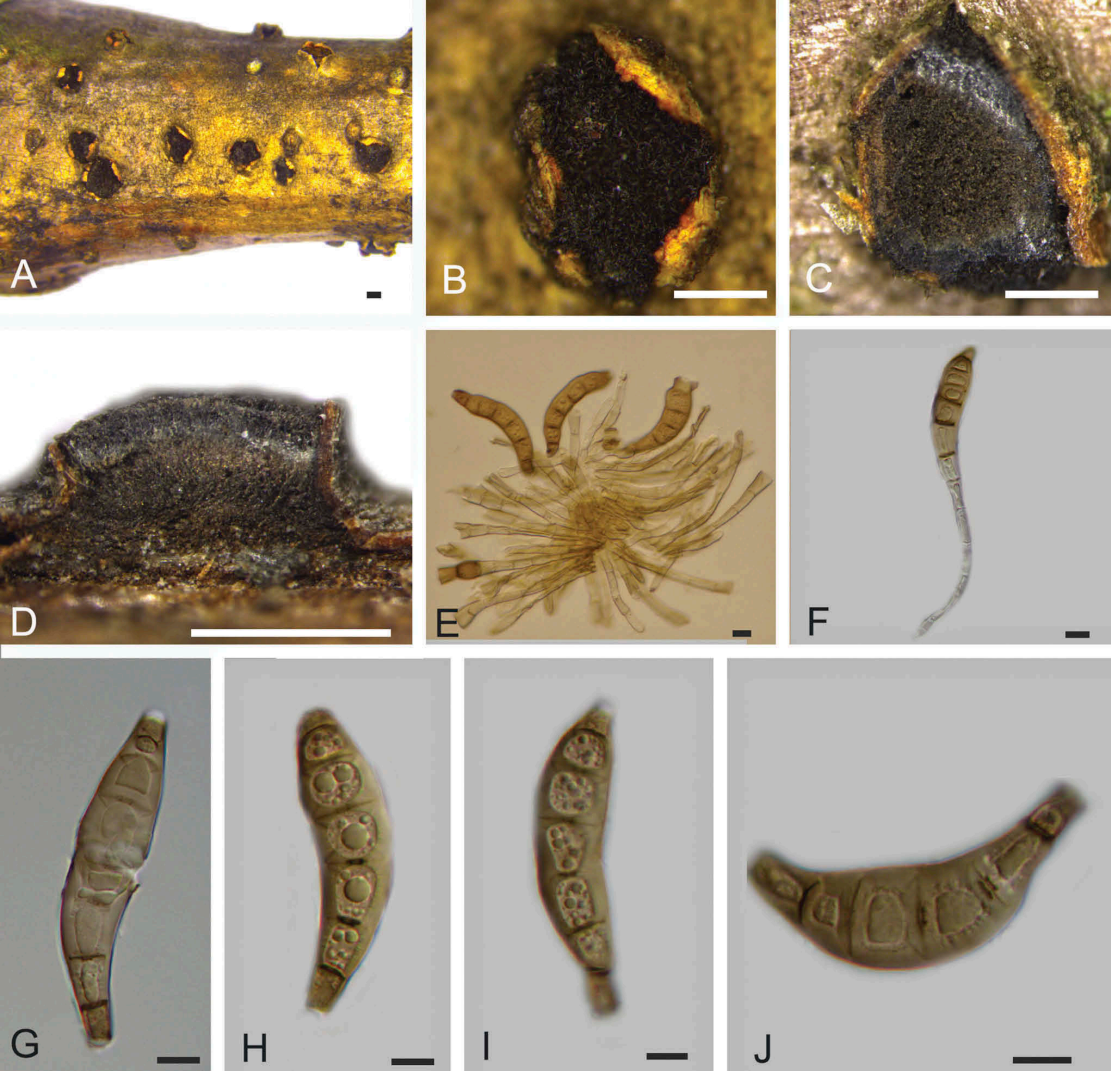
Morphology of *Coryneum sinense* from *Quercus serrata* (BJFC-S1421, holotype). A. B. Conidiomata on natural substrate in surface view. C. Transverse section through conidioma. D. Longitudinal section through conidioma. E, F. Conidiophores. G–J. Conidia. Bars: A–D = 0.5 mm; E–J = 10 μm.

***Coryneum sinense*** C.M. Tian, Voglmayr & N. Jiang, sp. nov. FIG. 6

MycoBank MB824595.

*Typification*: CHINA. SHAANXI PROVINCE: Shangluo City, Niubeiliang Reserve, 33°50′08.59″N, 109°18′57.36″E, 2208 m asl, on branches of *Quercus serrata, N. Jiang*, 7 Jul 2017 (**holotype** BJFC-S1421). Ex-type culture: CFCC 52452.

*Etymology: sinense* (Latin), named after China, where it was first collected.

*Sexual morph*: Not observed.

*Asexual morph*: Conidiomata acervular, 0.5–2.5 mm wide, 0.3–1.5 mm high ($\overset{ˉ}{x}$ = 1.4 × 0.8 mm, n = 20), solitary, erumpent through outer periderm layers of host, scattered, surface tissues above slightly domed. Conidiophores 40–100 μm long, 4–7 μm wide ($\overset{ˉ}{x}$ = 75 × 6 μm, n = 20), unbranched, cylindrical, septate, hyaline at apex, pale brown at base. Conidiogenous cells holoblastic, integrated, indeterminate, cylindrical, expanding toward apices, hyaline to pale brown, smooth, with 0–1 percurrent extensions. Conidia (50–)57–65(–76) × (13–)13.5–15(–17) μm, L/W = (3.3–)3.4–5.1(–5.9) (n = 50), slightly curved or not, broadly fusiform to clavate, dark brown, smooth-walled, 5–7-distoseptate, apical cell with a hyaline tip, truncate and black at base.

*Culture characters*: On PDA at 25 C, colonies growing slowly and symmetrically, reaching 50 mm diam within 30 d, becoming cinereous to dark gray in color with scant cottony aerial mycelium, asexual morphs developed after 40 d.

*Habitat and host range*: Dead corticated branches of *Quercus serrata*.

*Additional specimen examined*: CHINA. SHAANXI PROVINCE: Xiangxidong Garden, 32°40′32.51″N, 108°59′22.48″E, 2208 m asl, on branches of *Q. serrata, N. Jiang*, 7 Jul 2017 (BJFC-S1422; living culture CFCC 52453).

*Notes*: Species biodiversity of *Coryneum* is highest on the host genus *Quercus*. Muthumary and Sutton (1986) summarized eight species occurring on oak branches and published a key to *Coryneum* species on *Quercus. Coryneum sinense* differs from *C. arausiacum, C. depressum, C. elevatum, C. japonicum, C. megaspermum, C. megaspermum* var. *cylindricum, C. neesii, C. umbonatum*, and *C. quercinum* by unbranched conidiophores. In addition, conidial size and the number of distosepta also distinguish these species well (TABLE 1).

**Figure 7.**
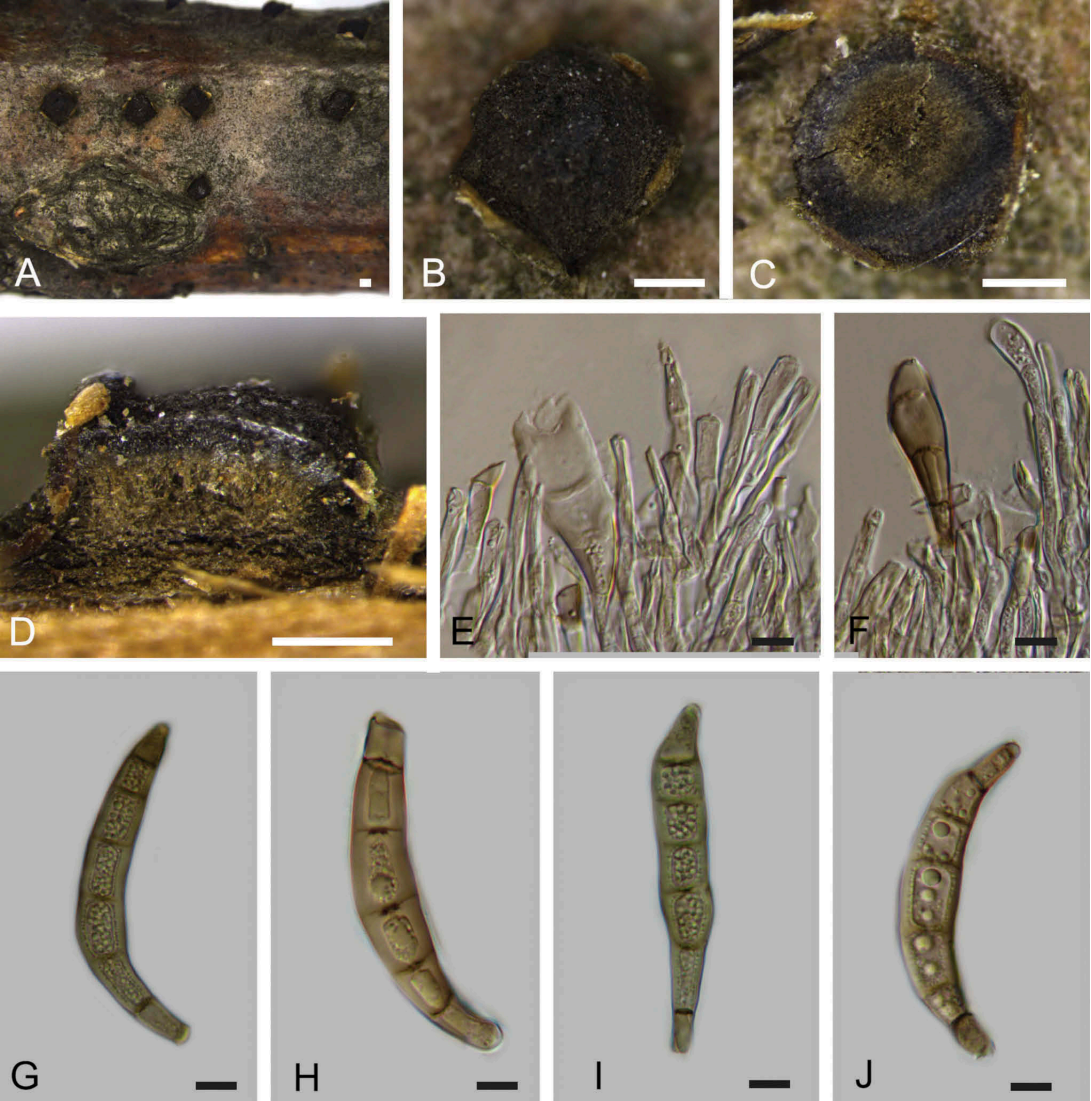
Morphology of *Coryneum suttonii* from *Castanea mollissima* (BJFC-S1423, ***holotype***). A, B. Conidiomata on natural substrate in surface view. C. Transverse section through conidioma. D. Longitudinal section through conidioma. E, F. Conidiophores. G–J. conidia. Bars: A–D = 0.5 mm; E–J = 10 μm.

***Coryneum suttonii*** C.M. Tian, Voglmayr & N. Jiang, sp. nov. FIG. 7

**Figure 8.**
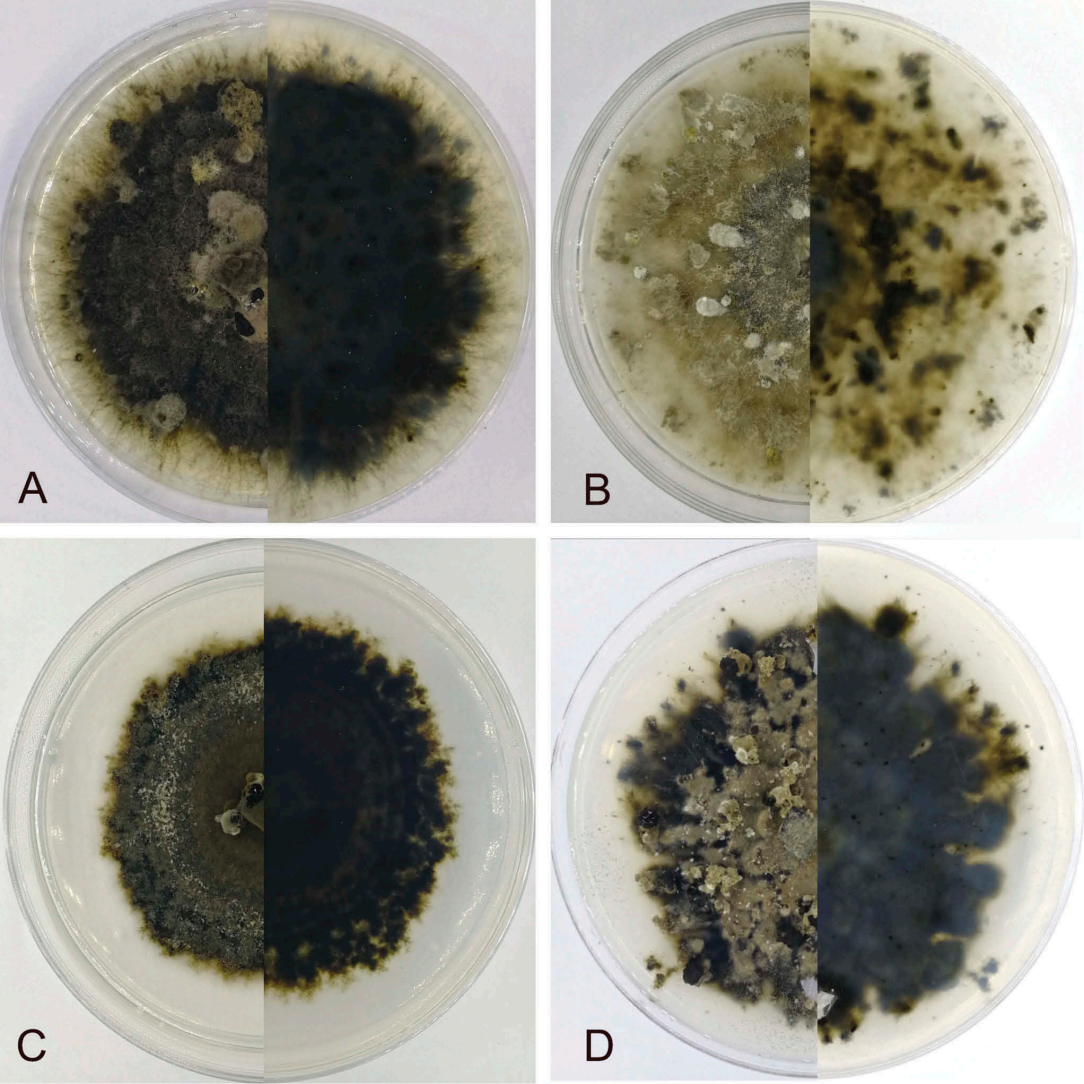
*Coryneum* cultures on PDA at 25 C after 30 d. A. *C. castaneicola*. B. *C. gigasporum*. C. *C. sinense*. D. *C. suttonii*.

MycoBank MB824596.

*Typification*: CHINA. SHAANXI PROVINCE: Shangluo City, chestnut plantation, 33°39′27.25″N, 109°07′15.48″E, 2504 m asl, on branches of *Castanea mollissima, N. Jiang*, 8 Jul 2017 (**holotype** BJFC-S1423). Ex-type culture: CFCC 52317.

*Etymology*: In honor of B. C. Sutton who published an extensive revision of *Coryneum* in 1975.

*Sexual morph*: Not observed.

*Asexual morph*: Conidiomata acervular, 0.5–2.0 mm wide, 0.3–1.2 mm high ($\overset{ˉ}{x}$ = 1.0 × 0.6 mm, n = 20), solitary, erumpent through outer periderm layers of host, scattered, surface tissues above slightly domed. Conidiophores 40–90 μm long, 4–8 μm wide ($\overset{ˉ}{x}$ = 70 × 6 μm, n = 20), unbranched, cylindrical, septate, hyaline at apex, pale brown at the base. Conidiogenous cells holoblastic, integrated, indeterminate, cylindrical, expanding toward apices, hyaline, smooth, with 0–1 percurrent extensions. Conidia (60–)68–74(–76) × (10–)10.5–13(–14.5) μm, L/W = (4.8–)6.4–6.5(–6.6) (n = 50), curved or not, fusiform to clavate, dark brown, smooth-walled, 4–5-distoseptate, apical cell with a hyaline tip, truncate and black at base.

*Culture characters*: On PDA at 25 C, colonies growing slowly and unevenly, reaching 60 mm diam within 30 d, becoming brownish gray to dark gray in color with scant cottony aerial mycelium, asexual morphs developed after 2 mo (FIG. 8).

*Habitat and host range*: Dead corticated branches of *Castanea mollissima.*

*Additional specimen examined*: CHINA. SHAANXI PROVINCE: Shangluo City, chestnut plantation, 33°39′27.25″N, 109°07′15.48″E, 2504 m asl, on branches of *C. mollissima, N. Jiang*, 8 Jul 2017 (BJFC-S1424, paratype; living culture CFCC 52318).

*Notes*: With the addition of two new species in the present publication, four *Coryneum* species now are known from chestnut trees (*Castanea* spp.). *Coryneum suttonii* can be distinguished from *C. gigasporum* by smaller conidia (60–76 × 10–14.5 μm in *C. suttonii* vs. 88–117 × 18–23 μm in *C. gigasporum*), and from *C. castaneicola* by fewer distosepta (4–5 in *C. suttonii* vs. 6–7 in *C. castaneicola*). The conidial length of *C. suttonii* is similar to that of *C. modonium* (60–76 μm in *C. suttonii* vs. 50–71 μm in *C. modonium*), but conidia of *C. suttonii* are distinctly narrower (10–14.5 μm in *C. suttonii* vs. 14–19 μm in *C. modonium*).

## DISCUSSION

The monotypic family Coryneaceae, with the genus *Coryneum*, was recognized as a separate group in Diaporthales in several studies (Voglmayr and Jaklitsch 2014; Senanayake et al. 2017; Voglmayr et al. 2017; Fan et al. 2018), although for most species in Coryneaceae DNA sequence data are lacking. In the present study, *C. castaneicola* was collected in China and both sexual and asexual morphs are described in detail. Three additional species, *Coryneum gigasporum* and *C. suttonii* from *Castanea mollissima* and *C. sinense* from *Quercus serrata*, are described as new based on morphology and ITS, 28S, *TEF1α*, and *RPB2* sequence data. The phylogenetic analyses (FIGS. 1, 2) also confirmed that the type species of *Coryneum* (*C. umbonatum*) and *Pseudovalsa* (*P. lanciformis*; syn. *C. lanciforme*) are closely related within the highly supported Coryneaceae and corroborated that the two genera are synonyms.

The results of the combined three-locus matrix (FIG. 2) confirmed that ITS-28S rDNA sequence data are insufficient to clearly resolve closely related species within *Coryneum*. This is consistent with other studies of Diaporthales (e.g., Voglmayr et al. 2012, 2017; Walker et al. 2012b; Voglmayr and Jaklitsch 2014), which reported a superior phylogenetic resolution of protein-coding markers such as *TEF1α* and *RPB2* compared with ITS-28S rDNA, reflecting the much stronger phylogenetic signal in the former. Therefore, in addition to ITS-28S rDNA, at least these markers should be routinely sequenced and included in phylogenetic studies of Diaporthales.

Most species of *Coryneum* occur on members of Fagaceae, specifically *Castanea* and *Quercus*. The most common host genus for *Coryneum* species is *Quercus*, with up to nine species and a variety recorded from oak. Muthumary and Sutton (1986) separated these species based on conidial size and number of distosepta. *Coryneum sinense* is different from all other known species from oaks in having unbranched conidiophores and unique conidial dimensions. Four species are now known from *Castanea*; they can be separated by conidial characters (TABLE 1). Following the extensive morphological investigations of Sutton (1975), species distinction based on conidial characters seems useful; however, this remains to be corroborated by DNA sequence data.

In our multigene analyses (FIG. 2), the different species from *Quercus* and *Castanea* hosts did not form a monophyletic group but were interspersed throughout the phylogram, indicating that speciation following host shifts is a common phenomenon in *Coryneum*, as it is for other genera of Diaporthales (e.g., Mejía et al. 2011b; Voglmayr et al. 2012, 2017;  Voglmayr and Jaklitsch 2014;Walker et al. 2014a, 2014b). We are uncertain whether or not the multiple specimens of the new species proposed in our study that originated from single plantations might be clones of the same genotype. For that reason, additional collecting trips were undertaken to examine the same hosts in different plantations in China from Apr to Jun 2018, but unfortunately our new species were not collected again. However, taxonomic sampling should be increased, both from *Quercus* and other host genera to investigate the evolution and speciation of the genus *Coryneum* in detail.
