# Supplementary material for: New species and records of Coryneum from China
Source: Mycologia. 2018 Nov 27;110(6):1172–88. doi: 10.1080/00275514.2018.1516969 (PMC6352375; doi:10.1080/00275514.2018.1516969)
Supplement: Supplemental Material [file UMYC_A_1516969_SM2516.pdf]

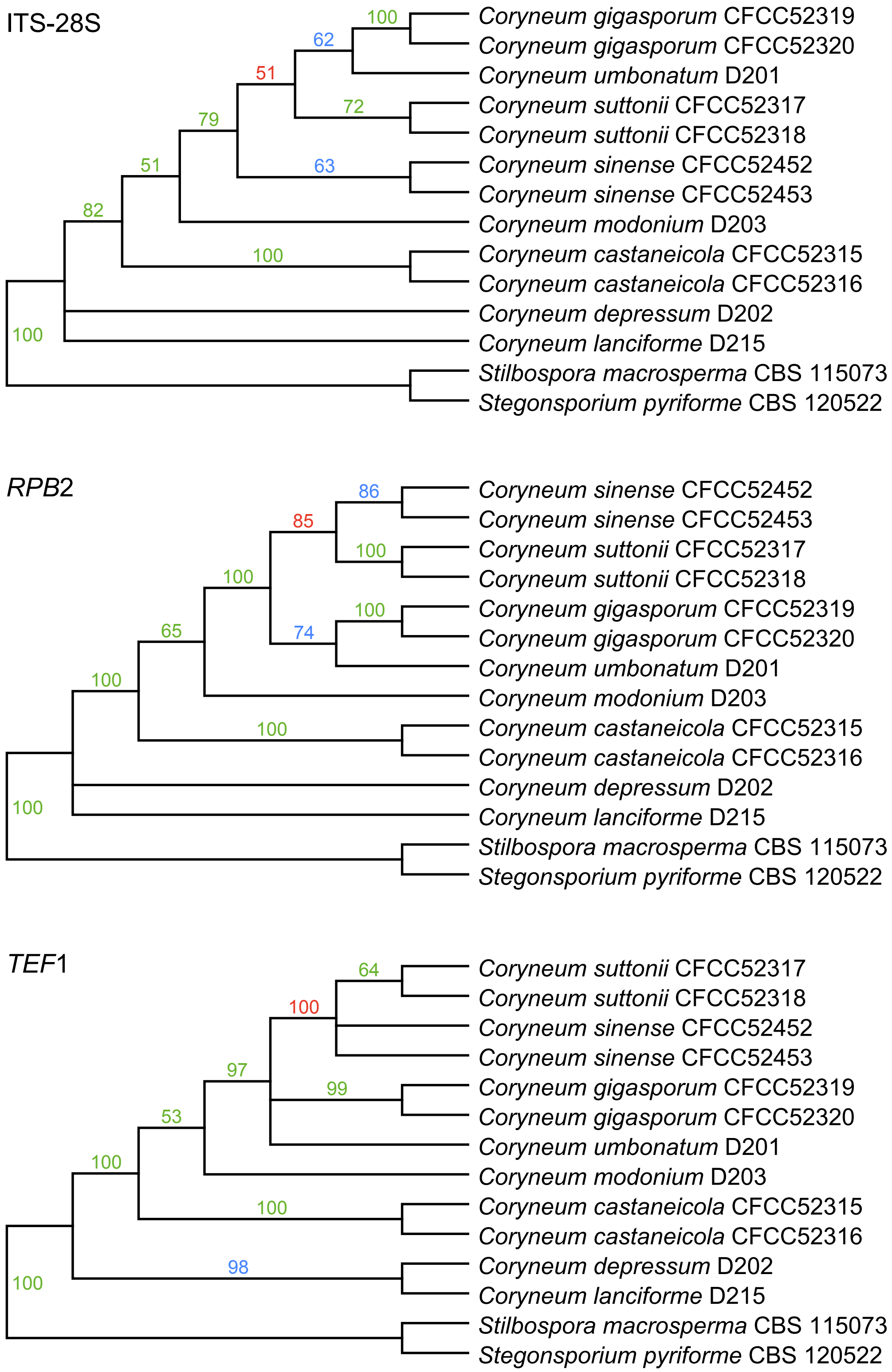

Supplementary Fig. 1. MP bootstrap trees of the ITS-28S, *RPB*2 and *TEF*1 matrices of *Coryneum* spp., obtained with PAUP (1000 bootstrap replicates, each with 10 rounds of heuristic search with random addition of sequences and subsequent TBR branch swapping; MULTREES option in effect, steepest descent option not in effect, COLLAPSE command set to MINBRLEN). Bootstrap support in green shows fully compatible topologies, in blue compatible topologies which do not receive support in all loci, and in red incompatible topologies. Tree topologies of the different loci were compatible, except for a moderately (85%, *RPB*2) to highly (100%, *TEF*1) supported *C. suttonii* - *C. sinense* clade versus a sister group relationship of the *C. sinense* clade to the *C. suttonii* - *C. umbonatum* - *C. gigasporum* clade in ITS-28S, which, however, is only poorly supported (51 %). The phylogenetic signal of the three loci is therefore considered to be congruent.
